# Dengue in Early Pregnancy: A Neglected Problem?

**DOI:** 10.7759/cureus.38740

**Published:** 2023-05-08

**Authors:** Ruchita Sinha, Mamta R Datta

**Affiliations:** 1 Obstetrics and Gynaecology, Tata Manipal Medical College, Jamshedpur, IND; 2 Obstetrics and Gynaecology, Tata Main Hospital, Jamshedpur, IND

**Keywords:** dengue, predictive factors, fetomaternal effects, abortion, pregnancy

## Abstract

Introduction

Dengue is caused by a virus from the Flaviviridae family. Although the literature on this disease is sparse, some studies have shown the effects of dengue in the first trimester of pregnancy. However, the sample size in these studies is limited.

Aims and objective

The current study aimed to compare foetomaternal outcomes in pregnant patients with dengue in early (< 24 weeks) and late (> 24 weeks) pregnancy and find the prevalence and predictive factors for abortion in pregnant patients with dengue.

Material and methods

This retrospective study included all pregnant patients (*n* *=* 62) admitted to the labour room over a period of six years from April 2016 to February 2022 and who were diagnosed with dengue anytime during pregnancy. Data were collected from their medical records and analysed. Differences between the two groups were assessed by the Chi-square test, Fisher’s exact test, and Mann-Whitney U test. A *p *value of less than 0.05 was considered significant.

Results

Out of a total of 62 patients, those with dengue at a gestational age of less than 24 weeks (*n* = 15) had more incidence of intrauterine growth restriction (55.6 vs 12.9%) (*p* value = 0.012) and oligohydramnios (66.7 vs 17.9%) (*p* value = 0.007). The incidence of abortion was 33.3%; among the patients at a gestational age of less than 12 weeks, 71.4% had an abortion. When the patients who had abortions were compared with those who did not, the factors predicting abortion were found to be a history of previous abortion (*p* value = 0.004), gestational age of less than 12 weeks (*p* value = 0.003), and decreased platelet count (*p* value = 0.03).

Conclusion

The effect of dengue infection in early pregnancy includes abortion, intrauterine growth restriction, and oligohydramnios, and these patients should be managed in a tertiary care hospital.

## Introduction

Dengue infections have undergone a rise in recent years and require extensive study [1]. Dengue is caused by a virus of the Flaviviridae family, transmitted by the infected Aedes mosquito. It has four serotypes: DEN-1, DEN-2, DEN-3, and DEN-4. DEN-3 and DEN-4 are Asian serotypes. Dengue virus can have grave consequences on pregnancy. There is sparse data about maternal and foetal effects of dengue infection and so managing these patients become difficult. In 2009, the World Health Organization (WHO) classified it as dengue without warning signs, with warning signs, and severe disease [1].

Dengue is characterised by three phases: 1. the febrile phase, characterised by fever and dehydration; 2. the critical phase, characterised by shock from plasma leakage, severe haemorrhage and organ impairment; and 3. the recovery phase, in which reabsorption from the extravascular component occurs and can cause hypervolemia if fluid therapy is excessive or extended into this period [2].

According to the WHO, spontaneous abortion is “the expulsion or extraction from its mother of an embryo or foetus weighing 500 g or less” [3]. Although the literature on this subject is sparse, a few studies show an association between dengue and abortion and other effects of dengue infection in the first trimester [4-6]. However, the number of patients in the susceptible group was low, hence the importance of this study. Furthermore, a few predictive factors for abortion were found in this study, which can be useful for these patients, and one is more careful. So, this study has the following objectives.

Primary objectives: 1. To study the clinical profile, the maternal and fetal outcomes in women infected with dengue fever in early pregnancy (< 24 weeks). 2. Comparison between the fetomaternal outcome in pregnant patients with dengue in early (<24 weeks) and late (> 24 weeks) pregnancy. Secondary objective: To find the prevalence and predictive factors for abortion in pregnant patients with dengue.

## Materials and methods

Study design

It is an observational retrospective study. It is done to determine the effect dengue has on early pregnancy. The study population was pregnant patients who were diagnosed with dengue during a period of five years from April 2016 to February 2022 at the tertiary hospital. Data were collected retrospectively from their medical records and analysed.

Maternal outcomes studied were - abortion, abruptio placentae, postpartum haemorrhage, presence of warning signs, dengue shock syndrome, and maternal mortality. Foetal outcomes studied were - intrauterine growth retardation, oligohydramnios, preterm labour, and stillbirth.

Study setting and study sample

The study is done at a tertiary hospital at Jamshedpur in Jharkhand, India. All pregnant patients who attended the hospital and were diagnosed with dengue during the time period of five years were included in the study. As a result, the total sample size was 62. Diagnosis of dengue was done by nucleic acid amplification test. It was done by detection of NS1 antigen and immunoglobulin (Ig)M antibody. NS1 is usually positive three days after onset of fever and IgM is positive after five days.

Statistical analysis

Continuous variables were expressed as means ± standard deviation and ranges. Categorical variables were expressed as numbers and percentages. Differences between the two groups were assessed by Chi-square or Fisher exact test as appropriate for categorical variables. Similarly, the Mann-Whitney U test was carried out for continuous variables. All tests were two-sided. A *p* value <0.05 was significant. The analysis was done using the SPSS version 21.0 software (IBM Corp., Armonk, NY).

The impact of dengue fever on mother and child in early pregnancy (<24 weeks) was studied and the prevalence of abortion in these patients was measured. Patients with dengue in early pregnancy (<24 weeks) were compared with patients with dengue in late pregnancy (>24 weeks) for fetomaternal outcomes. Patients who had abortions were compared with patients with a live pregnancy and predictive factors for abortion in these patients were assessed.

## Results

A total of 2402 dengue patients were admitted to the hospital during the study period, of which 62 (2.6%) were pregnant women, which was the study group. The sociodemographic profile of these patients is given in Table 1.

**Table 1:** Sociodemographic profile of patients

Demographic data	Number		Percentage
AGE GROUP (YEARS)			
18 – 25	10	16.1%	
26 - 35	35	56.45%	
36 – 40	9	14.5%	
> 40	8	12.9%	
EDUCATION LEVEL			
Below secondary	20	32.2%	
Secondary	30	48.4%	
University	12	19.4%	
OCCUPATION			
Housewife	46	74.2%	
Working	16	25.8%	
RESIDENCE			
Urban	38	61.3%	
Rural	24	38.7%	

Maximum patients were between 26 to 35 years of age, had completed a secondary level of education, were housewives, and resided in urban areas. Among all the pregnant patients, eight (12.9%) were in their first trimester, 17 (27.4%) in the second trimester, and 37 (69.3%) in the third trimester. Among the patients with dengue in early pregnancy, the mean age was 28.87 ± 5.08, 95% CI (24.83, 30.77), and gestational age was 14.20 ± 6.38, 95% CI (13.35, 21.25). Among the patients with dengue in late pregnancy, the mean age was 32.65 ± 6.97, 95% CI (29.82, 34.53), and gestational age was 32.08 ± 8.3, 95% CI (30.12, 37.60).

The patients with dengue in early pregnancy presented or had a history of the following symptoms: fever (seven, 46.6%), vomiting (four, 26.6%), epigastric pain (two, 20%), and headache (one, 6.6%). For the patients with dengue in late pregnancy, the presenting symptoms were fever (39, 100%), myalgia (30, 76.9%), headache (26, 66.6%), persistent vomiting (two, 5.5%), abdominal pain (one, 2.5%), and petechiae (one, 2.5%).

The patients with dengue in late pregnancy had lower platelet counts (92564.10 ± 388 vs 110777.78 ± 340) (*p* value = 0.435). Regarding the liver function profile, aspartate aminotransferase/alanine aminotransferase (AST/ALT) in patients with dengue in late pregnancy was higher (149.03/156.77) than in patients with dengue in early pregnancy (52.67/59.78). The difference in ALT was significant (*p* value = 0.048) (Table 2).

**Table 2:** Comparison between patients with dengue in early and late pregnancy IUGR: Intrauterine growth restriction; OLIGO: Oligohydramnios; PTL: Preterm labor; AST: Aspartate aminotransferase; ALT: Alanine aminotransferase; EFWT: Estimated foetal weight

Name of variables (n = 48)		Gestational age (n = 48)		p value
		< = 24 weeks (n = 09)	> 24 weeks (n = 39)	
IUGR N (%)	Yes	5 (55.6%)	5 (12.8%)	0.012
	No	4 (44.4%)	34 (87.2%)	
OLIGO N (%)	Yes	6 (66.7%)	7 (17.9%)	0.007
	No	3 (33.3%)	32 (82.1%)	
PTL N (%)	Yes	1 (11.1%)	2 (5.1%)	0.472
	No	8 (88.9%)	37 (94.9%)	
Stillbirth N (%)	Yes	0 (0.0%)	4 (10.3%)	1.00
	No	9 (100.0%)	35 (89.7%)	
Age	Mean ± SD	34.11 ± 4.16	32.31 ± 7.47	0.716
Platelet Count	Mean ± SD	110777.78 ± 34013.88	92564.10 ± 38886.34	0.435
AST	Mean ± SD	52.67 ± 37.53	149.03 ± 174.63	0.069
ALT	Mean ± SD	59.78 ± 31.32	156.77 ± 159.41	0.048
EFWT	Mean ± SD	2.35 ± 0.63	2.66 ± 0.51	0.209

Among the patients with dengue in early pregnancy (n = 15), five (33.3%) had an abortion (incidence in general population = 10%). Six patients (66.7%) developed oligohydramnios later in pregnancy (prevalence in general population = 4%), and five (33.3%) had the presence of warning signs. In the patients with dengue in later pregnancy (> 24 weeks) (n = 39), only seven (17.9%) developed oligohydramnios, and warning signs were present in seven (17.9%). Other complications in these patients were abruptio placenta (one, 1.7%); postpartum haemorrhage (five, 12.8%), which is high compared with the prevalence in the general population (2-4%); three (12.8%) developing dengue shock syndrome; and four (10.2%) deaths.

Dengue infection had adverse effects on the foetus as well. In the cases of dengue in early pregnancy (< 24 weeks), out of 15 cases, five (55.6%) had intrauterine growth restriction (IUGR). One (11.1%) had preterm delivery. In cases of dengue in the second half of pregnancy (>24 weeks), out of 39 patients, five (12.8%) developed IUGR and two (5.1%) had preterm labour. Four (10.3%) patients had a stillbirth. Therefore, IUGR was more prevalent among the patients with dengue in early pregnancy (*p* value = 0.012), and oligohydramnios was more prevalent (*p* value = 0.007) later in gestation, and it was statistically significant (Table 2).

Dengue and abortion

Fifteen patients had a gestational age of less than or equal to 24 weeks (26.3%). Among them, seven (46.6%) had a gestational age of less than 12 weeks. All the patients presented with fever and myalgia. Among these 15 pregnant patients who a gestational age of less than 24 weeks, five (33.3%) had an abortion. In patients with a gestational age of less than 12 weeks, five out of seven had an early abortion, with the percentage as high as 71.4%.

The characteristics of the patients who had an abortion (group A) were compared with those of the patients whose pregnancy was uneventful (group B). The average age of the patients whose pregnancy continued until term (group A) was 31 years, whereas the average age of the patients who had an abortion (group B) was 28 years. An important aspect that can be considered a predictive factor is the history of previous abortions. Four out of the five patients (80%) who had an abortion had a previous history of one or two abortions, whereas only two out of ten (20%) who had a successful pregnancy outcome had had previous abortions (*p* value = 0.017). Similarly, a correlation was found between the patients with low platelet counts and raised liver function tests. The average platelet count of the patients who had an abortion (group A) was 81,600 and that of group B was 1,13,00 (*p* value < 0.001). Similarly, elevated liver enzymes had a significant *p* value of < 0.001 (Table 3).

**Table 3:** Predictors of Abortion AST: Aspartate aminotransferase; ALT: Alanine aminotransferase

Name of variables (n = 15)		Group (n = 15)		*p* value
		Abortion (n = 05)	Non-Abortion (n = 10)	
Parity N (%)	A0	1 (20.0%)	0 (0.0%)	0.004
	A1	2 (40.0%)	0 (0.0%)	
	A2	1 (20.0%)	0 (0.0%)	
	A3	1 (20.0%)	0 (0.0%)	
	G1	0 (0.0%)	8 (80.0%)	
	G2	0 (0.0%)	1 (10.0%)	
	G3	0 (0.0%)	1 (10.0%)	
Age	Mean ± SD	31.00 ± 6.55	27.80 ± 4.16	0.440
Gestational age	Mean ± SD	8.0 ± 1.41	17.30 ± 5.51	0.003
Platelet count	Mean ± SD	90000 ± 17262.67	1130000 ± 27088.33	0.03
AST	Mean ± SD	56.00 ± 28.31	53.80 ± 34.30	0.594
ALT	Mean ± SD	77.00 ± 73.46	60.90 ± 27.78	0.859

Other factors, such as age and its impact on abortion, were also studied. Increased age (31.12 vs 28 years) was linked with a higher abortion rate, but the relation was not significant.

## Discussion

In a recent study by Brar et al., the miscarriage rate was 4.5% [4]. In another study conducted in Sri Lanka, the number of abortions observed was one at 24 weeks of gestation. It was a small study of 15 patients, and none were in the first trimester, which can be a cause of underreporting [5]. In a study by Basurko et al. with 53 patients, late miscarriage was found in two patients (3.8%). However, the authors did not exclude the confounding factor of gestational age, as patients in the third trimester are not at risk of abortion, which is why a low percentage was shown [6]. Although the exact cause is unknown, various studies have shown that it could be linked to hyperthermia, inflammatory response, and invasion of placental tissue by the virus [7-9].

The detrimental effect of hyperthermia on pregnancy is well known. Many research studies have shown that it causes congenital malformations and abortions in the first trimester. As stated by Graham et al., hyperthermia is a physical agent with a dose-response curve for abortions and malformations. The authors further state that there is a response by the embryo known as heat shock response, which protects it from the initial stages of hyperthermia. But if hyperthermia is not controlled, it can lead to congenital malformation and abortion [7]. A paper by Edwards et al. states that even mild exposure during the preimplantation period and more severe exposure during embryonic and foetal development often result in prenatal death and abortion [8].

Another theory proposes that the dengue virus causes an inflammatory response in the placenta and vertical transmission of the virus. As stated in a paper by Ribeiro et al., inflammatory responses include deciduitis, choriodeciduitis, intervillositis, focal and multifocal villitis, necrotizing villitis, proliferative villitis, and multifocal necrotizing villitis. They affect the growing embryo and cause abortion. Later in pregnancy, they can cause preterm birth [9].

Searching more literature, in another study, 5.2% of women who had an abortion showed recent dengue infection. Here, the study population is the number of patients who had an abortion [10]. Another study showed eight (32%) out of 25 women had an early miscarriage [11]. Finally, a study by Agarwal et al. showed that spontaneous abortion occurred for four of six women diagnosed with dengue in the first trimester and one in two women diagnosed in the second trimester [12].

Predictive factors for abortion

The two predictive factors that were observed were the previous history of abortions and the presence of thrombocytopenia. Pregnancy comprises a unique immunological condition to protect the foetus from maternal rejection, allowing adequate foetal development and protection against microorganisms [13,14]. In viral infections, there is the maternal immunological response, but in patients with recurrent abortions, the delicate immunological response is already disturbed, which is further altered by infection.

Another factor is thrombocytopenia, which probably causes an increased risk of haemorrhage between foetal membranes and decidua, leading to the disruption of pregnancy. The cause of thrombocytopenia in dengue is decreased bone marrow production, attenuation of megakaryocyte maturation, and increased destruction of platelets; hence there is an increased incidence of haemorrhage in these patients [15,16]. The average platelet count in patients with abortion was lower than in patients in which the pregnancy continued, with a significant *p* value.

This study has two limitations, which are the small sample size and its retrospective nature. However, it can be part of a larger analytical study which is the need of the hour.

## Conclusions

Dengue infection can affect pregnancy in any trimester, ranging from abortion in the first trimester to severe maternal illness in the second and third trimesters. While treating these patients with dengue in early pregnancy, we have to be aware of the high probability of these patients developing intrauterine growth restriction and oligohydramnios and should be treated in a tertiary hospital.

These patients have a higher risk of abortion (33.3%) and patients with the presence of predictive factors like thrombocytopenia and previous history of abortions should be careful. The knowledge of the association of dengue with abortion and its other fetomaternal effects is especially important in patients living in dengue-endemic areas like Andhra Pradesh, Arunachal Pradesh, Assam, and Bihar in India.

## References

[1] (2023). Dengue and severe dengue. https://www.who.int/news-room/fact-sheets/detail/dengue-and-severe-dengue.

[2] (2023). Dengue guidelines for diagnosis, treatment, prevention and control. https://apps.who.int/iris/handle/10665/44188.

[3] (1977). WHO: recommended definitions, terminology and format for statistical tables related to the perinatal period and use of a new certificate for cause of perinatal deaths. Modifications recommended by FIGO as amended October 14, 1976. Acta Obstet Gynecol Scand.

[4] Brar R, Sikka P, Suri V, Singh MP, Suri V, Mohindra R, Biswal M (2021). Maternal and fetal outcomes of dengue fever in pregnancy: a large prospective and descriptive observational study. Arch Gynecol Obstet.

[5] Kariyawasam S, Senanayake H (2010). Dengue infections during pregnancy: case series from a tertiary care hospital in Sri Lanka. J Infect Dev Ctries.

[6] Basurko C, Carles G, Youssef M, Guindi WE (2009). Maternal and fetal consequences of dengue fever during pregnancy. Eur J Obstet Gynecol Reprod Biol.

[7] Graham JM Jr, Edwards MJ, Edwards MJ (1998). Teratogen update: gestational effects of maternal hyperthermia due to febrile illnesses and resultant patterns of defects in humans. Teratology.

[8] Edwards MJ, Saunders RD, Shiota K (2003). Effects of heat on embryos and foetuses. Int J Hyperthermia.

[9] Ribeiro CF, Lopes VG, Brasil P, Pires AR, Rohloff R, Nogueira RM (2017). Dengue infection in pregnancy and its impact on the placenta. Int J Infect Dis.

[10] Tan PC, Soe MZ, Si Lay K, Wang SM, Sekaran SD, Omar SZ (2012). Dengue infection and miscarriage: a prospective case control study. PLoS Negl Trop Dis.

[11] Sondo KA, Ouattara A, Diendéré EA (2019). Dengue infection during pregnancy in Burkina Faso: a cross-sectional study. BMC Infect Dis.

[12] Agarwal K, Malik S, Mittal P (2017). A retrospective analysis of the symptoms and course of dengue infection during pregnancy. Int J Gynaecol Obstet.

[13] Mor G, Cardenas I (2010). The immune system in pregnancy: a unique complexity. Am J Reprod Immunol.

[14] PrabhuDas M, Bonney E, Caron K (2015). Immune mechanisms at the maternal-fetal interface: perspectives and challenges. Nat Immunol.

[15] Simmons CP, Farrar JJ, Nguyen vV, Wills B (2012). Dengue. N Engl J Med.

[16] de Azeredo EL, Monteiro RQ, de-Oliveira Pinto LM (2015). Thrombocytopenia in dengue: interrelationship between virus and the imbalance between coagulation and fibrinolysis and inflammatory mediators. Mediators Inflamm.

